# Nod1 Signaling Overcomes Resistance of S. pneumoniae to Opsonophagocytic Killing

**DOI:** 10.1371/journal.ppat.0030118

**Published:** 2007-08-24

**Authors:** Elena S Lysenko, Thomas B Clarke, Mikhail Shchepetov, Adam J Ratner, David I Roper, Christopher G Dowson, Jeffrey N Weiser

**Affiliations:** 1 Department of Microbiology, University of Pennsylvania School of Medicine, Philadelphia, Pennsylvania, United States of America; 2 Department of Pediatrics, University of Pennsylvania School of Medicine, Philadelphia, Pennsylvania, United States of America; 3 Department of Biological Sciences, University of Warwick, Coventry, United Kingdom; Children's Hospital Boston, United States of America

## Abstract

Airway infection by the Gram-positive pathogen Streptococcus pneumoniae (*Sp*) leads to recruitment of neutrophils but limited bacterial killing by these cells. Co-colonization by *Sp* and a Gram-negative species, Haemophilus influenzae (*Hi*), provides sufficient stimulus to induce neutrophil and complement-mediated clearance of *Sp* from the mucosal surface in a murine model. Products from *Hi,* but not *Sp,* also promote killing of *Sp* by *ex vivo* neutrophil-enriched peritoneal exudate cells. Here we identify the stimulus from *Hi* as its peptidoglycan. Enhancement of opsonophagocytic killing was facilitated by signaling through nucleotide-binding oligomerization domain-1 (Nod1), which is involved in recognition of γ-D-glutamyl-*meso-*diaminopimelic acid (*meso*-DAP) contained in cell walls of *Hi* but not *Sp*. Neutrophils from mice treated with *Hi* or compounds containing *meso*-DAP, including synthetic peptidoglycan fragments, showed increased *Sp* killing in a Nod1-dependent manner. Moreover, Nod1^−/−^ mice showed reduced *Hi*-induced clearance of *Sp* during co-colonization. These observations offer insight into mechanisms of microbial competition and demonstrate the importance of Nod1 in neutrophil-mediated clearance of bacteria in vivo.

## Author Summary

Pathogens are generally studied in the laboratory one species at a time. Most exist, however, in complex environments where they must adapt not only to their host but also to other members of the microbial flora. Using a mouse model of co-colonization, we have shown that one bacterial species (Haemophilus influenzae) can take advantage of the innate immune response of its host to outcompete and eliminate another species (Streptococcus pneumoniae) that resides in the same microenvironment of the upper respiratory tract. The molecular mechanism for this effect involves recognition of a cell wall fragment found on *H. influenzae,* but not on S. pneumoniae. The response to this immunostimulatory fragment requires Nod1, a host molecule that transmits inflammatory signals in response to specific peptides of the bacterial cell wall. This Nod1-mediated inflammatory stimulation triggers an increase in the ability of a type of white blood cell (neutrophil) to engulf and then kill *S. pneumoniae,* effectively removing it from its niche on the mucosal surface of the host airway. Our study, therefore, provides a demonstration of the importance of Nod1 in neutrophil-mediated clearance of bacterial infection. In addition, we have described a mechanism for interspecies competition between microbes that occurs through selective stimulation of host innate immune responses.

## Introduction

Successful pathogens have mechanisms both to avoid triggering inflammatory responses and/or to evade the inflammatory response they induce in their host. In the case of the Gram-positive Streptococcus pneumoniae (*Sp*), a major pathogen of the human respiratory tract, infection involving normally sterile parts of the airway is characterized by acute inflammation with a marked and brisk recruitment of neutrophils [1]. This neutrophil influx, however, is often insufficient to clear the infection until type-specific antibody promotes opsonophagocytic killing. Before such antibody is generated, pneumococci are relatively resistant to neutrophil-mediated killing even when opsonized by complement [2]. The inability of phagocytes to eliminate pneumococci in this period may account for the rapid and often overwhelming progression of pneumococcal pneumonia, a disease responsible for more than a million deaths a year [3]. In fact, in experimental acute pneumonia, neutrophils enhance the likelihood of death without impacting bacterial clearance [4]. Likewise, in a murine model of carriage, intranasal inoculation of *Sp* induces recruitment of neutrophils into the nasal spaces, yet systemic depletion of neutrophils has little effect on the density of colonizing bacteria [5,6]. In contrast, when co-colonized with the Gram-negative respiratory tract bacteria Haemophilus influenzae (*Hi*), the neutrophil influx is sufficient to rapidly clear *Sp* from the mucosal surface [6]. Clearance during co-colonization is not seen if either neutrophils or complement are systemically depleted, indicating that killing occurs through neutrophil-mediated phagocytosis of *Sp* opsonized by complement. These in vivo observations demonstrate that one microbe can co-opt the innate immune response of the host to prevail over a competitor that resides within a similar niche. Enhanced killing of *Sp* can be modeled *ex vivo* using neutrophils derived from peritoneal exudates cells (PECs) treated in vivo with *Hi* or its products. Thus, components of *Hi* are sufficient to stimulate neutrophil activity that overcomes the resistance of complement-opsonized *Sp* to phagocytic killing.

The focus of this study is to define the mechanism leading to effective neutrophil-mediated killing of *Sp* that occurs in the absence of specific antibody. We observed that peptidoglycan fragments from *Hi* are sufficient to promote neutrophil-mediated phagocytosis of opsonized *Sp*. Pathways for the recognition of and response to peptidoglycan fragments leading to NF-κB-dependent transcriptional activation and pro-inflammatory responses have been partially characterized [7]. Peptidoglycan fragments containing the minimal structure γ-D-glutamyl-*meso-*diaminopimelic acid (*meso*-DAP) found in Gram-negative bacteria, including *Hi,* act through a cytoplasmic signaling molecule, nucleotide-binding oligomerization domain-1 (Nod1) [8–10]. In the peptidoglycan of most Gram-positive bacteria, including *Sp, meso*-DAP is replaced by lysine, a structural difference of a single carboxyl group that is sufficient to prevent effective signaling involving Nod1 [11]. In addition, another peptidoglycan fragment, muramyl dipeptide (MDP), common among most Gram-negative and Gram-positive bacteria, is the minimal structure needed for responses involving a separate cytoplasmic immune signaling molecule, Nod2 [12]. Our findings provide a demonstration of the contribution of Nod1-mediated signaling to the anti-bacterial activity of neutrophils and their ability to clear mucosal infection.

## Results

### Killing of *Sp* Occurs via Opsonophagocytosis

The increased ability of *ex vivo* PECs to kill *Sp* when elicited following intraperitoneal (i.p.) administration of heat-killed *Hi* (HK*Hi*) allowed us to examine the mechanism whereby one species stimulates the killing of another. When HK*Hi*-stimulated PECs were divided by density gradient centrifugation into mononuclear cell– and neutrophil-containing fractions, only the neutrophil-enriched fraction demonstrated killing of *Sp* (unpublished data). This result correlated with the absence of killing by HK*Hi*-stimulated PECs when elicited from mice depleted of neutrophils by prior treatment with RB6-8C5, an antibody to murine Ly6.G [6,13]. Addition of HK*Hi* correlated with increased neutrophil activation as confirmed by increased expression of the marker Mac-1(complement receptor 3 [CR3], CD11b/CD18) in cells co-expressing Ly6.G [6]. Moreover, increased killing of *Sp* following administration of HK*Hi* was observed with neutrophil-enriched PECs derived from parental but not congenic Mac-1^−/−^ mice (Figure 1). This finding pointed to the requirement of complement-mediated opsonization for neutrophil recognition. When heat-inactivated serum or serum from C3^−/−^ mice was used as a complement source, no killing by HK*Hi*-stimulated neutrophil enriched PECs was seen, confirming the requirement of active complement. Although C3 may be activated by either classical or alternative pathways, killing in the presence of serum from *scid* mice lacking antibody made it less likely that complement was being activated by the classical pathway [6]. The requirement for the alternative pathway was confirmed by showing a lack of *Sp* killing when serum from factor B–deficient mice was used as a complement source (Figure 1). Thus, results using PECs indicated that products of *Hi* stimulate neutrophil-mediated phagocytic killing of *Sp* opsonized primarily by activation of the alternative pathway of complement.

**Figure 1:**
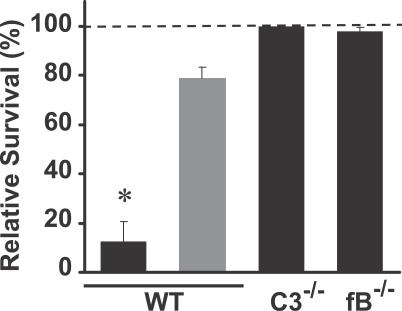
*Hi*-Enhanced Killing of *Sp* Requires Opsonization Involving the Alternative Pathway of Complement and Recognition by Mac-1 (CD11b/CD18) Neutrophil-enriched PECs were obtained from mice following i.p. administration of heat-inactivated whole *Hi*636 in casein. The effect of HK*Hi* on the ability of neutrophil-enriched PECs to kill *Sp*1121 is shown relative to controls where neutrophil-enriched PECs were elicited by casein administration alone. Survival of *Sp*1121 was assessed over a 45-min incubation with complement in serum from wild-type mice (WT), C3-deficient mice (C3^−/−^), or factor B–deficient mice (fB^−/−^) as indicated. Neutrophil-enriched PECs were obtained from Mac1^−/−^ (grey bar) or control C57Bl/6 (black bars) mice. No stimulation of killing was observed in controls using heat-inactivated complement or cells from animals pretreated with monoclonal antibody RB6-8C5 to deplete neutrophils. Values represent ≥ three independent determinations in duplicate ± SD. *p < 0. 01 compared to other conditions.

### Enhancement of Opsonophagocytic Killing Is Independent of TLR2, TLR4, and rPAF

Products of *Hi* have previously been shown to signal pro-inflammatory responses through toll-like receptor (TLR) 2 and TLR4, through recognition of its lipopolysaccharide (LPS) and lipoproteins, respectively [14,15]. In addition, platelet-activating factor receptor (rPAF)-mediated signaling has been described for those *Hi* phase variants expressing the cell surface ligand phosphorylcholine [16]. Opsonophagocytic killing was assessed in neutrophil-enriched PECs derived from TLR2^−/−^ mice. These showed increased killing in response to HK*Hi* and were as active as cells derived from the TLR2-expressing mouse strain (Figure 2). Opsonophagocytic killing was also compared in neutrophil-enriched PECs derived from CH3/OuJ and C3H/HeJ mice, which express functional and non-functional TLR4, respectively. TLR4 did not contribute to *Sp* killing in response to HK*Hi* stimulation. Moreover, HK*Hi* derived from isogenic mutants expressing or not expressing phosphorylcholine stimulated similar levels of *Sp* killing by neutrophil-enriched PECs (unpublished data) [17]. Together, these results showed that the enhancement of opsonophagocytic killing occurs independently of non-redundant signaling involving known cell surface pattern recognition receptors for *Hi*, including TLR2, TLR4, and rPAF.

**Figure 2:**
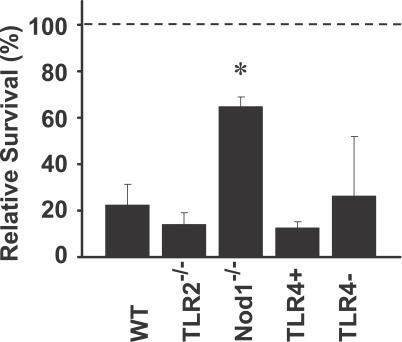
*Hi*-Enhanced Opsonophagocytic Killing of *Sp* Requires Nod1 Neutrophil-enriched PECs were obtained from C57Bl/6 mice (WT) or congenic mice lacking TLR2 or Nod1. C3H mice with functional or non-functional TLR4 were also compared. The effect of i.p. administration of heat-inactivated whole *Hi*636 in casein on the ability of neutrophil-enriched PECs to kill *Sp*1121 is shown relative to controls where neutrophil-enriched PECs were elicited by casein administration alone. Values represent ≥ three independent determinations in duplicate ± SD. *p < 0.05 compared to other groups of the same genetic background.

### Enhancement of Opsonophagocytic Killing through Recognition of Peptidoglycan

This unexpected finding led us to characterize the signal from *Hi* that enhances the opsonophagocytic killing of *Sp*. Neither lysis of HK*Hi* by sonication, nor prior treatment with proteinase K, diminished stimulation of killing by neutrophil-enriched PECs, which indicates the involvement of a non-proteinaceous bacterial product. However, there was no stimulation of neutrophil-enriched PECs by purified LPS (in doses up to 50 μg/animal) extracted from *Hi* or Escherichia coli (Figure 3). These findings were also consistent with a signaling pathway other than recognition of *Hi* components by TLR2, TLR4, or rPAF.

**Figure 3:**
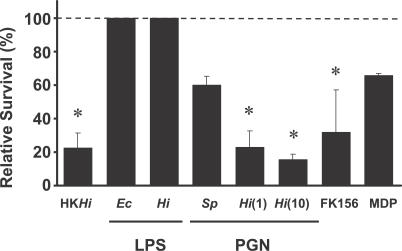
The Ability of Bacterial Components to Stimulate Opsonophagocytic Killing of *Sp* Neutrophil-enriched PECs were obtained from C57Bl/6 mice pretreated by i.p. administration of casein containing heat-inactivated whole *Hi*636 (HK*Hi, n* = 6), E. coli LPS (50 μg/animal, *n* = 3), *Hi* LPS (50 μg/animal, *n* = 4), *Sp* peptidoglycan (PGN, 10 μg/animal, *n* = 5), *Hi* peptidoglycan (PGN, 1.0 or 10 μg/animal, *n* = 4 and 3, respectively), FK-156 (1.0 μg/animal, *n* = 6), or MDP (10 μg/animal, *n* = 3). Survival of *Sp*1121 is shown relative to controls where neutrophil-enriched PECs were elicited by casein administration alone. Values represent duplicate determinations of the number of independent experiments indicated above for each condition ± SD. *p < 0.01 compared to casein alone control.

In contrast, purified *Hi* peptidoglycan at a dose as low as 1 μg/animal was sufficient to stimulate increased killing of *Sp* by neutrophil-enriched PECs (activity equivalent to 10^7^ HK*Hi*). Purified peptidoglycan from *Sp* (or Staphylococcus aureus) was less active even when administered at a 10-fold higher dose (Figure 3 and unpublished data). The greater potency of *Hi* peptidoglycan correlated with the stimulation of killing by HK*Hi* but not HK*Sp* [6]. This observation indicated that structural differences between cell wall fragments of these species may be an important determinant of their peptidoglycan-mediated signaling. To confirm this hypothesis, FK-156, a synthetic muropeptide containing *meso*-DAP, was tested and showed a level of stimulatory activity equivalent to purified *Hi* peptidoglycan when administered at an equivalent concentration. Experiments with FK-156 also demonstrated that *Hi* peptidoglycan could provide a sufficient stimulus to neutrophil-enriched PECs that accounts for their enhanced killing of *Sp* and makes it unlikely that a contaminant in the peptidoglycan preparation could explain our findings. In contrast, MDP at the equivalent concentration was a relatively poor stimulus.

### Requirement for Nod1 in Enhancement of Opsonophagocytic Killing and Interspecies Competition

The potency of *Hi* peptidoglycan, as well as that of FK-156, suggested that stimulation of opsonophagocytic killing involved recognition of *Hi* components by Nod1. In order to examine this possibility, neutrophil-enriched PECs from Nod1^−/−^ mice were analyzed for their response to HK*Hi* and FK-156. As predicted, administration of FK-156 (10 μg/animal) stimulated *Sp* killing by cells in parental, but not in Nod1^−/−^ mice (Figure 4A). Neutrophil-enriched PECs from Nod1^−/−^ mice also showed a diminished response to HK*Hi,* demonstrating that Nod1 accounts for a significant proportion of the signaling generated by innate recognition of this organism.

**Figure 4:**
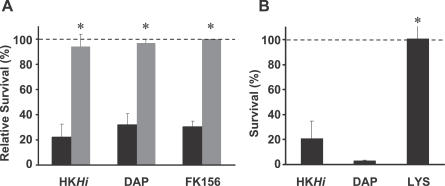
The Ability of Synthetic Peptidoglycan Fragments to Stimulate Opsonophagocytic Killing of *Sp* (A) Neutrophil-enriched PECs were obtained from C57Bl/6 (black bars) or congenic Nod1^−/−^ (grey bars) mice pretreated by i.p. administration of casein containing heat-inactivated whole *Hi*636 (HK*Hi*), murNAcTRI_DAP_ (DAP, 10 μg/animal), or its synthetic analog, FK-156 (10 μg/animal). Survival of *Sp*1121 is shown relative to controls where neutrophil-enriched PECs were elicited by casein administration alone. Values represent ≥ three independent determinations in duplicate ± SD. *p < 0.01 compared to the same treatment in parental mice. (B) Neutrophil-enriched PECs were obtained from C57Bl/6 mice pretreated by i.p. administration without casein of heat-inactivated whole *Hi*636 (HK*Hi*), murNAcTRI_DAP_ (DAP, 10 μg/animal), or murNAcTRI_LYS_ (LYS, 10 μg/animal). Values represent ≥ three independent determinations in duplicate ± SD. *p < 0.01 compared to other groups.

To further confirm this observation, a *meso*-DAP-containing peptide, murNAcTRI_DAP_, was synthesized using the Mur enzymes of Gram-negative bacteria [18]. As predicted, its ability to stimulate *Sp* killing by neutrophil-enriched PECs was equivalent to that of FK-156 and dependent on Nod1 (Figure 4A). In contrast, a synthetic form of the corresponding lysine-containing tripeptide found in *Sp* peptidoglycan, murNAcTRI_LYS_, lacked stimulatory activity at the same concentration. In Figure 4B, the effect of these peptides on killing is compared in neutrophil-enriched PECs elicited with peptide in buffer alone without the addition of casein to show that administration of murNAcTRI_DAP_ is sufficient and murNAcTRI_LYS_ is insufficient to enhance killing of *Sp*.

Our findings using neutrophil-enriched PECs stimulated in vivo and tested in killing assays *ex vivo* suggested that co-colonization with *Hi* in competition experiments with *Sp* should promote clearance of *Sp* in a Nod1-dependent manner. Indeed, a significant decrease in the density of *Sp* colonization was observed in Nod1^+/+^ but not in Nod1^−/−^ mice co-infected with *Hi* (Figure 5). The reduced interspecies competition in the absence of Nod1 demonstrated an important role for peptidoglycan recognition in the innate response to Gram-negative bacteria on the mucosal surface.

**Figure 5:**
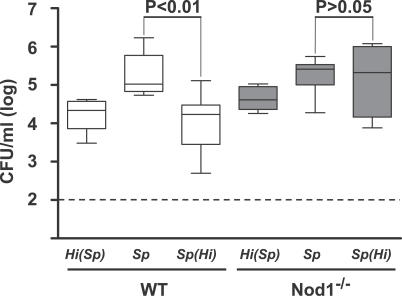
Effect of Nod1 on Competition between Species during Co-Colonization The density of *Sp*1121 (*Sp*) in upper respiratory tract lavage was determined at 24 h post-intranasal inoculation with *Sp* alone or together with *Hi*636 (*Hi*) in Nod1^−/−^ or parental C57Bl/6 mice (WT). Box-and-whiskers plot indicates high and low values, median and interquartile ranges; *n* ≥ 10 mice from three independent experiments in each group. Co-inoculated species shown in parentheses. The lower limit of detection for bacteria in lavage culture was 10^2^ CFU/ml (indicated by a dashed line).

### Mechanism of Enhanced Neutrophil-Mediated Opsonophagocytosis Involving Nod1

Next, we explored the mechanism for increased opsonophagocytic killing stimulated through Nod1. Levels of the proinflammatory chemokine MIP-2, which functions as a murine neutrophil attractant and activator, were previously shown to correlate with neutrophil influx into the nasal spaces [6]. MIP-2 levels increased in response to co-colonization, but were not significantly different between co-colonized Nod1^−/−^ and parental mice (Figure 6A). Analysis of tissue sections from co-colonized mice, both Nod1^−/−^ and parental, showed an intimate association of both *Sp* (and *Hi*) with neutrophils in the lateral nasal spaces (Figure 6B). These results suggested that the recruitment of neutrophils and their migration to mucosal sites with bacteria were not affected by the expression of Nod1 in this model. Additional evidence that Nod1 did not impact neutrophil migration came from comparisons by flow cytometry of PECs elicited by HK*Hi* or FK-156 (Figure 6C). No difference between Nod1^−/−^ and parental mice was seen in the proportion of total cells expressing Ly6.G (neutrophils). For both Nod1^−/−^ and parental mice, Ly6.G positive cells also expressed the markers CD18/CD11b (activated neutrophils).

**Figure 6:**
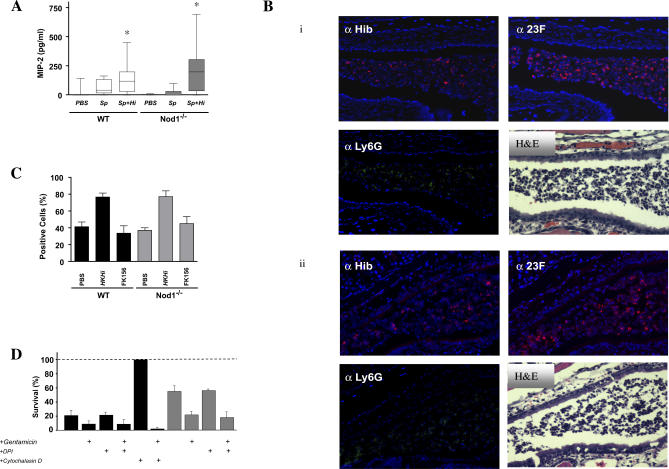
Contribution of Nod1 to Chemokine Production, Neutrophil Recruitment, and Phagocytosis (A) The effect of Nod1 on stimulation of macrophage inhibitory protein (MIP-2) in upper respiratory tract lavage fluid in response to *Sp* and co-colonization with *Hi* in Nod1^−/−^ or parental C57Bl/6 mice (WT). Box-and-whiskers plot indicates high and low values, median and interquartile ranges; *n* ≥ 13 mice from two independent experiments for each co-infected group. *p < 0.02 compared to PBS control. (B) Effect of Nod1 on the neutrophil influx in response to bacteria following co-colonization. Immunofluorescence showing co-localization of *Hi* and *Sp* with neutrophils in the murine nasal spaces. Parental (i) or Nod1^−/−^ (ii) mice were co-colonized with *Hi*636 and *Sp*1121, and at 24 h post-inoculation, adjacent 5-μm frozen parasagittal tissue sections through the lateral nasal spaces of the same animal were stained with anti-capsular polysaccharide serum specific to type b *Hi* (αHib), type 23F *Sp* (α23F), or αLy6.G antibody to mouse neutrophils. Hematoxylin and eosin–stained parasagittal sections (H&E) show the neutrophil influx into the lumen between adjacent nasal turbinates. DAPI nuclear staining (blue). Magnification 200×. (C) FACS analysis of the effect of Nod1 of the influx of activated neutrophils. Administration (i.p.) of casein with 10^6^ heat-inactivated *Hi* or FK-156 (1.0 μg/animal) was compared to casein alone for the effect on PECs expressing Ly6.G and the activation marker CD11b/CD18. Values represent the percent of total PECs expressing both markers and are means of three independent determinations ± SD. (D) Killing of *Sp* by neutrophil-enriched PECs at a ratio of one bacterium to 25 effector cells obtained from C57Bl/6 (black bars) or congenic Nod1^−/−^ (grey bars) mice pretreated by i.p. administration of heat-inactivated whole *Hi*636 (HK*Hi*). Where indicated, activated neutrophils were treated *ex vivo* with cytochalasin D, an inhibitor of actin polymerization, or DPI, an inhibitor of NADPH oxidase and the oxidative burst. Survival of intracellular *Sp* was based on bacterial counts following treatment with gentamicin sulfate. Values represent ≥ three independent determinations in duplicate ± SD.

We next considered whether Nod1 signaling affected uptake or killing of bacteria. Gentamicin sulfate was added at the end of killing assays to determine the proportion of *Sp* surviving within neutrophils as a measure of phagocytic activity and killing. Comparison of neutrophil-enriched PECs elicited by HK*Hi* from Nod1^+/+^ or Nod1^−/−^ mice showed no difference in the proportion of viable intracellular bacteria (Figure 6D). Preincubation of neutrophil-enriched PECs with cytochalasin D, to inhibit actin rearrangements and block phagocytosis, resulted in minimal survival after gentamicin treatment. These findings confirmed the role of phagocytosis in neutrophil-mediated killing and suggested that Nod1 signaling did not affect the uptake of *Sp*. Killing of *Sp* by neutrophil-enriched PECs elicited by HK*Hi* from Nod1^+/+^ or Nod1^−/−^ mice was also not affected by pretreatment with dibenziodolium chloride (DPI), a blocker of the oxidative burst. Together, these observations suggest that Nod1 signaling acts on events following phagocytosis on a non-oxidative pathway for killing *Sp*.

## Discussion

Although numerous studies have defined Nod1-mediated effects of bacteria or their cell wall products in vitro, our understanding of its contribution to innate immune responses to bacterial infection in vivo remains limited (reviewed in [7,19]). We demonstrate here that the Nod1 signaling pathway can respond to *meso*-DAP-containing compounds to increase clearance of *Sp* from the mucosal surface of the airway. Thus, Nod1 was shown to be important in dictating the outcome of competition between two pathogens that occupy a similar niche in their host [6]. Enhanced killing of *Sp* required products from another organism, since cell wall fragments from *Sp,* like most Gram-positive species, do not signal through Nod1. Our findings are relevant to polymicrobial infection and situations in which products from multiple types of organisms are present. This information adds to our previous report, which describes how combinations of microbes and microbial products synergize to enhance inflammatory responses [20]. Mucosal surfaces, in particular, are generally colonized simultaneously with multiple species. The paradigm of one species promoting an innate immune response that affects a competitor may be underappreciated, because most models of infection typically examine responses to individual microbial species.

While our model was useful in revealing a role for Nod1 in vivo, it also demonstrates that bacteria that succeed in such environments must have mechanisms to evade its clearance-promoting effects. The specificity for bacterial cell wall components that act through Nod1 suggests a mechanism whereby many Gram-positive pathogens that lack *meso*-DAP may avoid signaling events that lead to neutrophil-mediated killing. Likewise, the density of colonizing *Hi* during co-infection was not affected by Nod1 signaling, in contrast to clearance of *Sp* during co-colonization. This suggests that *Hi* may be resistant to the response induced by its *meso*-DAP-containing peptidoglycan, and also to the enhancement of opsonophagocytic killing by neutrophils seen against *Sp*. In addition to the synthesis of stem peptides without *meso*-DAP, there may be multiple mechanisms to evade peptidoglycan recognition and stimulation of immune signaling through Nod1 [21]. For example, it has recently been reported that modification of the α-carboxylic acid group of iso-glutamic acid, the residue proximal to *meso*-DAP, to an amide diminishes signaling through Nod1 and may be a mechanism for immune invasion by some pathogens [22].

Both *Sp* and *Hi* are considered extracellular pathogens, which are unable to effectively access intracellular pathways [23]. In the case of epithelial cells, pore-forming toxins or delivery via the type IV pilus have been shown to be necessary for peptidoglycan to gain access to the cytoplasm [24,25]. Moreover, Nod1-deficient mice were shown to be more susceptible to infection by Helicobacter pylori expressing the cag pathogenity island type IV secretion apparatus than were wild-type mice [25]. Our observation in this report that peptidoglycan fragments alone are sufficient to induce Nod1-dependent effects shows that access to these cytoplasmic pathways may not be similarly limited for professional phagocytes. In killing assays, however, bacteria or peptidoglycan fragments were delivered in vivo and their activity tested *ex vivo*. Thus, we cannot confirm whether the effect of injected compounds or bacterial products on neutrophil function was direct. Attempts to treat neutrophils in vitro with immunostimulatory fragments that are active when provided in vivo were not sufficient to elicit a direct effect in killing assays. It is unlikely that this is due to a lack of Nod1 expression in these cells, because in contrast to other members of the Nod protein family, Nod1 expression is ubiquitous [7]. It remains possible that Nod1-mediated signaling requires other cell types, such as epithelial cells, and that its effects on neutrophil function are indirect.

A further consideration is that neutrophils have cell wall–degrading enzymes, such as lysozyme, that may generate more biologically active peptidoglycan fragments. This could account for the effects of purified peptidoglycan in our study, which contrasts with prior reports where only synthetic products are active. Thus, both the processing of peptidoglycan and the ability of cell wall fragments to access the cytoplasm may be important factors for signaling events involving neutrophils. In this regard, it has been suggested that *Sp* and other Gram-positive pathogens synthesize modified peptidoglycan that is resistant to lysozyme [21,26,27]. Thus, a number of adaptations may contribute to minimizing Nod-mediated signaling by Gram-positive bacteria despite their greater quantity of peptidoglycan per cell.

Our study demonstrates that the resistance of *Sp* to killing by neutrophils (Figure 4B) can be overcome by a specific immune signaling pathway. Findings in this study with microbial products and synthetic *meso*-DAP-containing peptidoglycan fragments add to a prior report that systemic administration of FK-156 enhanced host resistance to various microbial infections [28]. Bacterial killing in our system required opsonization, which for *Sp* strain *Sp*1121 occurred through activation of the alternative pathway of complement, followed by phagocytosis by activated, Mac-1 (CR3, CD11b/CD18)-expressing neutrophils. One of the ligands of Mac-1, or CR3, is iC3b [29]. It remains unclear how Nod1-mediated signaling enhances Mac-1-dependent opsonophagoytic killing of complement-opsonized *Sp*. It has been suggested that Nod1 transduces signals that can stimulate chemokine production and neutrophil recruitment [30]. We did not observe, however, a Nod1-related effect on the increase in MIP-2 levels or influx of neutrophils into either the peritoneal cavity or the nasal spaces in response to bacteria. Likewise, no effect of Nod1 on the uptake of bacteria or generation of an oxidative burst was detected. Rather, killing of *Sp* in our model resulted from stimulation of non-oxidative activity of neutrophils. Reduced killing in the presence of inhibitors of actin polymerization and rearrangement, and the requirement for complement, argue against Nod1-mediated enhancement of previously described mechanisms for extracellular killing of *Sp* by neutrophils [31]. We are currently characterizing this oxidative burst–independent anti-pneumococcal effect of neutrophils and the contribution of Nod1 to stimulation of this biological activity.

Findings in this study also show a limited role of other signaling pathways in clearance of *Sp* from the mucosal surface of the murine airway. *Sp* has previously been shown to activate cellular NF-κB-dependent immune responses through Nod2. However, the effect of fragments acting through Nod2, including MDP, purified *Hi* or *Sp* peptidoglycan, and live or killed *Hi* or *Sp*, was minor in comparison to those acting through Nod1 [6,32]. Moreover, the *Hi*-induced increase in *Sp* killing by neutrophil-enriched PECs was not influenced by the pathogen-associated molecular pattern receptors, TLR2 or TLR4, in a non-redundant manner. Thus, our study provides an example where the predominant signaling response of the innate immune system to a bacterial challenge appears to be through Nod1.

## Materials and Methods

### Bacterial strains and culture conditions.

*Hi* and *Sp* strains were grown as previously described [33]. Strains used in vivo were selected because of their ability to colonize efficiently the murine nasal mucosa and included *Hi*636 (a type b capsule-expressing, spontaneously streptomycin-resistant mutant of *Hi* strain Eagan), and *Sp*1121 (a type 23F capsule-expressing *Sp* isolate from the human nasopharynx [34]). Genetically modified *Hi* mutants of strain Eagan that constitutively express or lack phosphorylcholine on its LPS were previously described [17].

### Mouse strains.

Six-week-old mice used in the study were housed in accordance with Institutional Animal Care and Use Committee protocols. Mouse strains included C57Bl/6J and congenic Nod1^−/−^ (Millennium Pharmaceuticals, http://www.mlnm.com/), B6.129S4-Itgam^tm1Myd^/J (Jackson Laboratories, http://www.jax.org/), and TLR2^−/−^ (provided by H. Shen, University of Pennsylvania). Mac-1 (CD11b/CD18)-deficient mice (Jackson Laboratories) have a targeted mutation in the gene for integrin alpha M or CR3 [35]. Neutrophils from these animals are deficient in phagocytosing complement-opsonized particles and in several Fc-mediated functions. The genotype of Nod1^−/−^ (CARD4-deficient) mice was confirmed by PCR using primers CARD4-F2 (5′-CTTAGGCATGACTCCCTCCTGTCG-3′), CARD4-R1 (5′-GATCTTCAGCAGTTTAATGTGGGAGTGAC-3′), and CARD4-RB (5′-CCATTCAAGCTGCGCAACTGTTG-3′). Sequences and PCR protocols were supplied by Charles River Laboratories Genetic Testing Services (http://www.criver.com/), where the colony was derived. TLR2^−/−^ mice have a targeted disruption of the gene encoding the C-terminus of the extracellular domain of TLR2 and display an increased susceptibility to bacterial infections [36]. Serum was also obtained from factor B–deficient and C3-deficient mice (provided by J. Lambris, University of Pennsylvania) [37,38]. TLR4-sufficient and -deficient mice were obtained from Jackson Laboratories. C3H/HeJ (TLR4-deficient) mice have a spontaneous mutation that occurred in wild-type C3H/HeOuJ (TLR4-sufficient) mice at an LPS response locus (mutation in TLR4 gene), making C3H/HeJ mice resistant to endotoxin [39].

### Mouse model of nasal colonization.

Mice were used in a previously described model of nasal colonization with *Sp* and *Hi* [34]. Briefly, groups of at least ten mice per condition were inoculated intranasally with 10 μl containing 1 × 10^7^ CFU of PBS-washed, mid-log phase *Hi, Sp,* or both applied separately to each naris. Then, 24 h post-inoculation, the animal was sacrificed, the trachea cannulated, and 200 μl of PBS instilled. Lavage fluid was collected from the nares for determination of viable counts of bacteria in serial dilutions plated on selective medium containing antibiotics to inhibit the growth of contaminants (100 μg/ml streptomycin to select for *Hi*636, and 20 μg/ml neomycin to select for *Sp*1121).

### Isolation and characterization of murine neutrophils.

Neutrophil-enriched PECs were isolated as previously described [40]. Briefly, phagocytes were obtained by lavage of the peritoneal cavity (8 ml/animal with PBS containing 20 mM EDTA) of mice treated 24 h and again 2 h prior to cell harvest by i.p. administration of 10% casein in PBS (1 ml/dose). Administration of casein provided for a higher and more consistent yield of cells. Cells collected from the peritoneal cavity lavage (PECs) were enriched for neutrophils or monocytic cells using separation by a Ficoll density gradient centrifugation according to the manufacturer's protocol (MP Biomedicals, http://www.mpbio.com/). Neutrophil or monocytic cell-enriched fractions were collected and washed with 5 ml of Hank's buffer without Ca^++^ or Mg^++^ (GIBCO, http://www.invitrogen.com/) plus 0.1% gelatin. An aliquot of these cells was characterized using FACS for staining of granulocytes with anti-mouse Gr-1 mAb to Ly6.G (BD Biosciences, http://www.bdbiosciences.com/) and showed >90% positively stained cells following enrichment. Additional characterization involved staining for CD11b/CD18 (BD Biosciences). Where indicated, heat-inactivated *Hi* (*Hi*636), bacterial components, FK-156 (an analog of *meso*-DAP provided by Astellas Pharmaceuticals, http://www.us.astellas.com/), or synthetic peptidoglycan fragments were co-administered intraperitoneally with or without the casein solution as indicated. PBS-washed, mid-log phase bacteria (10^7^ cells/animal) were heat-inactivated by treatment at 65 °C for 30 min and shown to be non-viable.

### Phagocytic killing assays.

Neutrophil-enriched PECs were counted by trypan blue staining and adjusted to a density of 7 × 10^6^ cells/ml. Killing during a 45-min incubation at 37 °C with rotation was assessed by combining 10^2^ PBS-washed, mid-log phase bacteria (in 10 μl) with complement source (in 20 μl), 10^5^ mouse phagocytes (in 40 μl), and Hank's buffer with Ca^++^ and Mg^++^ (GIBCO) plus 0.1% gelatin (130 μl). Earlier time points and fewer effector to target cells were shown in pilot experiments to result in less killing. The complement source consisted of fresh mouse serum from C57Bl/6 mice unless indicated otherwise. After stopping the reaction by incubation at 4 °C, viable counts were determined in serial dilutions. Percent killing was determined relative to the same experimental condition without i.p. administration of bacterial products or FK-156 (casein alone). For groups without co-administered casein, the percent killing was calculated by comparison to controls with inactivated complement (56 °C for 30 min) where there was no loss of bacterial viability. Additional controls consisting of heat-inactivated *Hi*636 administered without casein gave similar levels of killing, confirming that killing was stimulated by bacterial products rather than by casein.

Where indicated, neutrophils were preincubated with 10 μM DPI, an NADPH-ubiquinone oxidoreductase inhibitor, for 15 min at 37 °C. The respiratory burst of activated neutrophils and its inhibition by DPI was assessed by cytochrome C oxidation with activation by treatment with 25 nM phorbol 12-myristate 13-acetate (PMA; Sigma, http://www.sigmaaldrich.com/) as a control. To inhibit phagocytosis, neutrophils were pretreated with cytochalasin D (20 μM, Sigma) for 15 min at 37 °C. Intracellular pneumococci were quantified using viable counts following the addition of gentamicin sulfate (final concentration 300 μg/ml). After a 20-min incubation at 37 °C, the antibiotic was removed by serial washing prior to plating for viable counts.

### Isolation of peptidoglycan and other bacterial components.

*Hi* LPS was purified by hot-phenol extraction from strain Eagan as previously described [41]. E. coli LPS and Staphylcoccus aureus peptidoglycan were purchased from Sigma.

Preparation of peptidoglycan from *Hi* was modified from a previously described protocol [42]. Briefly, strain *Hi*636 was grown overnight in sBHI, pelleted at 6,000*g* at 4 °C, and washed with Tris-buffered saline (TBS). The pellet was resuspended in 5 ml of cold dH_2_O, and cells were lysed in boiling SDS (5%) for 30 min. Lysates were collected at 150,000*g,* resuspended in dH_2_O, and washed once with TBS. Glycogen and nucleic acids were removed by treatment with α-amylase (Fluka 10070, from Bacillus subtilis) and DNAse/RNAse A (Sigma) for 2 h at 37 °C, followed by overnight incubation at 37 °C with agitation and 100 μg/ml of trypsin (Worthington Biochemical, http://www.worthington-biochem.com/) in the presence of 10 mM CaCl_2_. To stop the reaction, 10 mM EGTA was added and the peptidoglycan preparation was boiled in 5% SDS for 30 min. After extensive washing, *Hi* peptidoglycan was lyophilized and resuspended at 5 mg/ml in endotoxin-free water.

For preparation of peptidoglycan from *Sp,* bacteria were grown in tryptic soy medium and treated as above, except cells were also treated in 0.5% Na-layrilsarcosin prior to boiling in SDS (5%) [43]. *Sp* peptidoglycan was additionally treated with hydrofluoric acid (49% for 48 h at 4 °C with agitation) to remove teichoic acid as described [44]. The pellet was washed extensively with dH_2_O, twice with acetone, and lyophilized.

### Preparation of peptidoglycan fragments.

N-acetlymuramyl-L-alanyl-γ-D-glutamyl-meso-2,6-diaminopimelic acid (murNAcTRI_DAP_), N-acetlymuramyl-L-alanyl-γ-D-glutamic acid (murNAcDI), and N-acetlymuramyl-L-alanyl-γ-D-glutamyl-L-lysine (murNAcTRI_LYS_) were prepared as described previously [18,45,46]. Briefly, recombinant Pseudomonas aeruginosa (*Pa*) MurA, MurB, MurC, and MurD were used to synthesise uridine 5′diphosphoryl-N-acetlymuramyl-L-alanyl-γ-D-glutamic acid (UDP-murNAcDI); additionally, *Pa* MurE was used to synthesise uridine 5′diphosphoryl-N-acetlymuramyl-L-alanyl-γ-D-glutamyl-meso-2,6-diaminopimelic acid (UDP-murNAcTRI_DAP_), and *Sp* MurE was used to synthesise uridine 5′diphosphoryl-N-acetlymuramyl-L-alanyl-γ-D-glutamyl-L-lysine (UDP-murNAcTRI_LYS_). Electrospray ionization mass spectrometry (negative ion) was used to confirm the molecular weight of synthesized compounds. Purity was assessed by analytical anion exchange chromatography using a GE Healthcare Mono Q HR5/5 column (http://www.gelifesciences.com/) and by continuous spectrophotometric enzyme assay with MurE for UDP-murNAcDI, and MurF for UDP-murNAcTRI_DAP_ and UDP-murNAcTRI_LYS_. N-acetlymuramyl-peptides were produced by mild acid hydrolysis (0.1 M HCl, 100 °C, 1 h) of the corresponding uridine 5′diphosphoryl-N-acetlymuramyl-peptides. Complete hydrolysis was confirmed by continuous spectrophotometric enzyme assay with MurE for murNAcDI (MDP), and MurF for murNAcTRI_DAP_ and murNAcTRI_LYS_. Peptides were analysed by electrospray ionisation mass spectrometry (positive ion). The concentration of other hydrolysis products (UDP, UMP, and P_i_) was established by continuous spectrophotometric enzyme assay. Corresponding concentrations of UDP, UMP, and P_i_ were added to the casein-only control.

### Histology and immunofluorescence.

At 24 h post-inoculation, the animal was sacrificed and decapitated, and the head was fixed for 48 h in 4% paraformaldehyde in PBS. The head was then decalcified by serial incubations in 0.12 M EDTA (pH 7.0) at 4 °C over 1 mo before freezing in Tissue-Tek O.C.T. embedding medium (Miles, Elkhart, Indiana, United States) in a Tissue-Tek Cryomold. Then, 5-μm-thick sections were cut, air dried, and stored at −80 °C. Frozen-imbedded tissue sections were stained with hematoxylin and eosin (H&E) following a 10-min fixation step in 10% neutral buffered formalin (NBF). Sections were then dehydrated in alcohol, cleared in xylene, and mounted in cytoseal (Richard-Allan Scientific, http://www.rallansci.com/). Immunofluorescent staining on frozen tissue was performed and visualized as previously described [47]. Neutrophil-like cells were stained using rat anti-mouse Ly6G mAb (BD Biosciences) followed by anti-rat Ig secondary antibody [6]. To detect *Sp*1121, sections were incubated with antisera to *Sp* type 23F (Statens Serum Institut, http://www.ssi.dk/) followed by anti-rabbit Ig secondary antibody. To detect *Hi*636, sections were incubated with antisera to *Hi* type b (DIFCO Laboratories, http://www.bd.com/ds/) followed by anti-rabbit Ig secondary antibody.

### Measurement of MIP-2 concentration.

Upper respiratory tract lavage fluid was assayed for the concentration of macrophage inhibitory protein (MIP-2) by ELISA in duplicate according to the manufacturer's instructions (Pharmingen, http://www.bdbiosciences.com/).

### Statistical analysis.

Statistical comparisons of colonization among groups were made by the Kruskal–Wallis test with Dunn's post-test (GraphPad Prism 4; GraphPad Software, http://www.graphpad.com/). In vitro killing assays were compared by ANOVA with Tukey post-tests as appropriate.

## Supporting Information

### Accession Number

The GenBank (http://www.ncbi.nlm.nih.gov/Genbank/index.html) accession number for murine Nod1 is NM_172729.

## Abbreviations

CR3: complement receptor 3
DPI: dibenziodolium chloride
*Hi*: *Haemophilus influenzae*
HK*Hi*: heat-killed *Haemophilus influenzae*
i.p.: intraperitoneal
LPS: lipopolysaccharide
MDP: muramyl dipeptide
*meso-*DAP: γ-D-glutamyl-*meso*-diaminopimelic acid
Nod1: nucleotide-binding oligomerization domain-1
PEC: peritoneal exudates cell
*Sp,*: *Streptococcus pneumoniae*
